# Donor-Derived Cell-Free DNA: Attractive Biomarker Seeks a Context of Use

**DOI:** 10.3389/ti.2023.12406

**Published:** 2023-12-01

**Authors:** Angelica Pagliazzi, Oriol Bestard, Maarten Naesens

**Affiliations:** ^1^ Nephrology and Renal Transplantation Research Group, Department of Microbiology, Immunology and Transplantation, KU Leuven, Leuven, Belgium; ^2^ Kidney Transplant Unit, Nephrology Department, Vall d’Hebron University Hospital, Barcelona, Spain; ^3^ Department of Nephrology and Renal Transplantation, University Hospitals Leuven, Leuven, Belgium

**Keywords:** donor-derived cell-free DNA, post-transplant monitoring, context of use, predictive values, clinical utility

## dd-cfDNA, a Promising Biomarker

The search for biomarkers for clinical use in kidney transplant monitoring sometimes seems a tantalizing torment.

**FIGURE 1:**
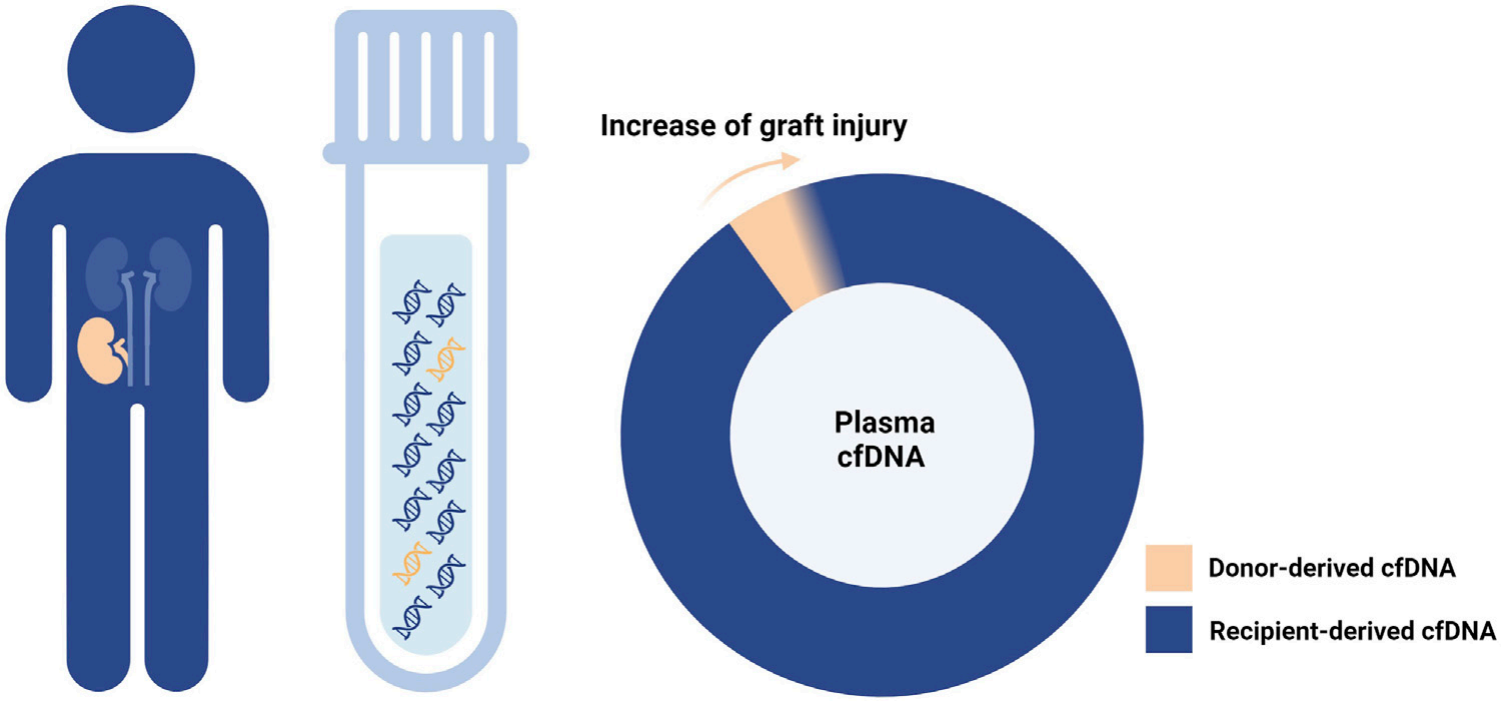
Donor-derived cell-free DNA and graft injury. As the graft damage advances, cell-free DNA (cfDNA) is increasingly released from the graft tissue into the recipient plasma.

The first hint about the potential application of donor-derived cell-free DNA (dd-cfDNA) in post-transplant monitoring dates back 25 years [1]. Specifically released by donor tissue cells (graft cells or donor hematopoietic cells residing within the graft) mainly at the time of cell death, dd-cfDNA is tightly linked to the graft status and, therefore, a promising non-invasive biomarker (Figure 1).

The advancement of more comprehensive and scalable DNA sequencing technologies, coupled with the easy accessibility and short half-life of cfDNA, has paved the way for the development of commercially available assays for measuring dd-cfDNA in the plasma of transplanted recipients. In the past 10 years, after demonstration of analytical robustness, these assays have been validated in clinical practice for kidney transplant recipients, and consistently demonstrated a significant correlation between plasma dd-cfDNA levels and graft damage [2–5].

These promising findings have led to the clinical adoption of dd-cfDNA assays for monitoring the occurrence of graft rejection and injury in kidney transplant recipients in the Unites States. This was boosted by Medicare reimbursement in 2017, and positive coverage decisions from several commercial payers. In Europe, however, the adoption of dd-cfDNA assays in clinical practice lags behind, due to cost concerns of the test and a lack of data demonstrating their clinical utility and context of use.

## Diagnostic Value of dd-cfDNA in European Context

In two prospective single-center studies in this journal, Benning et al. and Mantios et al. report on the diagnostic performance of dd-cfDNA% in discriminating kidney transplant recipients experiencing graft rejection from rejection-free patients, at the time of clinically indicated biopsies [6, 7]. The optimal discriminative threshold for dd-cfDNA% was consistent across the two studies, underlining the analytic robustness of the assay. The overall dd-cfDNA% performance in discriminating rejection from no rejection was greater for full-blown rejection diagnoses (Antibody-mediated rejection (AMR) vs. no rejection Area under the ROC curve (AUC) 0.90; T cell-mediated rejection (TCMR) vs. no TCMR AUC 0.73) than for borderline changes (borderline vs. no rejection AUC 0.66), in accordance with previous studies [6].

A major added value of the study by Benning et al [6] is the reporting on negative (NPV) and positive predictive values (PPV), essential parameters to identify the best clinical context of use of a test [8]. The authors conclude that dd-cfDNA% might help in clinical decision making, warranting, or excluding the need of a kidney transplant biopsy in recipients at higher-risk of graft rejection, i.e., when the clinician decides to perform an indication biopsy based on other blood and/or urine biomarkers.

## Can Biopsies Be Safely Avoided With dd-cfDNA Testing?

However, a NPV of 77% with the best cut-off (0.57%) is far from excluding all rejection cases. As outlined by the authors, the lower sensitivity of dd-cfDNA for borderline changes, could be a major downside of the test at time of graft dysfunction. Borderline changes in indication biopsies are considered as clinically meaningful [9] and are also treated similarly like TCMR by the majority of centers. How reassured can one be by testing negative for severe rejection with dd-cfDNA%, and how safely can a biopsy be omitted, when clinically meaningful borderline changes and sometimes even TCMR are not detected with the test and proposed threshold?

Instead of proposing single thresholds, more work is needed to identify the thresholds below which rejection (including borderline changes) can be safely excluded, and to calculate how many biopsies could be avoided with such test. This would allow for establishing the true clinical benefit of dd-cfDNA% testing at the time of clinical suspicion of injury/rejection and help calculate cost-effectiveness in such a context.

## Non-Specificity of dd-cfDNA, and Detection of Subclinical Injury

In addition, these two studies [6, 7] highlight other aspects that remain to be untangled on this topic.

First, while typically higher in severe active rejection, dd-cfDNA% shows considerable variability within specific rejection categories, correlation with both active and chronic lesions, and possibly increased levels in case of rejection-free graft injuries (such as calcineurin inhibitor toxicity or acute tubular injury). These observations suggest that although dd-cfDNA may be used as an intuitive biomarker of graft injury, what exactly is being measured at the biological level has not yet been elucidated. Coupling plasma dd-cfDNA and biopsy gene expression data, a weak association between dd-cfDNA and injury as well as atrophy-fibrosis gene sets was noted. This supports the idea that dd-cfDNA correlates with unspecific parenchymal injury and not primarily with alloimmune mediated inflammation [10].

Such non-specificity of dd-cfDNA for graft rejection is not necessarily a disadvantage *per se*. By being more comprehensive, such non-invasive biomarker could indicate invasive confirmation of a potentially treatable condition, in addition to rejection. Nonetheless, larger prospective studies, including heterogeneous real-life kidney transplant populations and integrating multiple layers of information (detailed demographic, clinical, serological, virological, and histological data, activity and chronicity indices, blood, and biopsy omics data) are needed to untangle dd-cfDNA biology in renal allograft recipients and eventually extend the applicability of dd-cfDNA testing in post-transplant monitoring.

Second, beyond its value at time of clinical suspicion by avoiding some biopsies, timely detection of subclinical and/or incipient immunological activation is an even greater unmet need in post-transplant monitoring. Besides protocol biopsies, which cannot be performed as a serial testing approach, there are not many other options available for frequent surveillance of kidney transplant status and identification of subclinical rejection or graft injury [11, 12]. Whether dd-cfDNA% has sufficient diagnostic performance in such specific context of use remains to be studied. Important here will be the false positive rate (and herewith related PPV). When PPV is too low (few true positive cases in the test positive group), this could lead to anxiety and performance of more non-informative biopsies, instead of less.

## Conclusion

In conclusion, the graft-specificity and apparent intuitive use of dd-cfDNA% resulted in an acceleration of its clinical implementation in the United States. Despite a large body of research been done and slowly advancing insights into its added value, many questions and confusion remains, hindering more global implementation of dd-cfDNA% as biomarker in kidney transplantation. We must remain critical and focus on intrinsic biology and the best context of use. Especially the latter will be very important for reimbursement discussions with payers in, e.g., European countries.

Moving from a promising biomarker to a widely used standard biomarker goes through larger prospective studies in real-life patient populations, and even randomized trials with clinically meaningful endpoints, such as the number of biopsies that can be avoided with non-invasive monitoring. This requires tireless efforts to integrate current monitoring practice with the results of dd-cfDNA measurements in a wide range of clinical scenarios.

Unlike Tantalus, who is eternally close to food and water without ever reaching them, we are rapidly closing the knowledge gap around dd-cfDNA testing for kidney transplantation. Well-conducted studies evaluating clinical utility and context of use are needed to implement dd-cfDNA testing in routine clinical care in Europe.

## Data Availability

The original contributions presented in the study are included in the article, further inquiries can be directed to the corresponding author.
